# Effect of Structure on the Interactions between Five Natural Antimicrobial Compounds and Phospholipids of Bacterial Cell Membrane on Model Monolayers

**DOI:** 10.3390/molecules19067497

**Published:** 2014-06-06

**Authors:** Stella W. Nowotarska, Krzysztof J. Nowotarski, Mendel Friedman, Chen Situ

**Affiliations:** 1 Institute for Global Food Security, School of Biological Sciences, Queen’s University Belfast, David Keir Building, Stranmillis Road, Belfast BT9 5AG, UK; E-Mail: snowotar@hotmail.co.uk; 2 School of Biological Sciences, Queen’s University Belfast, Medical Biology Centre, 97 Lisburn Road, Belfast BT9 7BL, UK; E-Mail: krzysztof.nowotarski@coventry.ac.uk; 3 Agricultural Research Service, United States Department of Agriculture, Western Regional Research Center, Albany, CA 94710, USA; E-Mail: mendel.friedman@ars.usda.gov

**Keywords:** natural antimicrobials, phospholipid monolayers, langmuir balance, isotherm, surface potential, antimicrobial mechanisms

## Abstract

Monolayers composed of bacterial phospholipids were used as model membranes to study interactions of the naturally occurring phenolic compounds 2,5-dihydroxybenzaldehyde and 2-hydroxy-5-methoxybenzaldehyde, and the plant essential oil compounds carvacrol, cinnamaldehyde, and geraniol, previously found to be active against both Gram-positive and Gram-negative pathogenic microorganisms. The lipid monolayers consist of 1,2-dihexadecanoyl-*sn*-glycero-3-phosphoethanolamine (DPPE), 1,2-dihexa- decanoyl-*sn*-glycero-3-phospho-(1'-*rac*-glycerol) (DPPG), and 1,1',2,2'-tetratetradecanoyl cardiolipin (cardiolipin). Surface pressure–area (π-A) and surface potential–area (Δψ-A) isotherms were measured to monitor changes in the thermodynamic and physical properties of the lipid monolayers. Results of the study indicated that the five compounds modified the three lipid monolayer structures by integrating into the monolayer, forming aggregates of antimicrobial –lipid complexes, reducing the packing effectiveness of the lipids, increasing the membrane fluidity, and altering the total dipole moment in the monolayer membrane model. The interactions of the five antimicrobial compounds with bacterial phospholipids depended on both the structure of the antimicrobials and the composition of the monolayers. The observed experimental results provide insight into the mechanism of the molecular interactions between naturally-occurring antimicrobial compounds and phospholipids of the bacterial cell membrane that govern activities.

## 1. Introduction

Natural compounds of plant origin have been known for their effective antimicrobial activity since ancient time [1] and are still widely used for treatment and prevention of infections by millions of people in many parts of the world. Recent studies attempt to combine both screening of bioactive compounds from natural sources and investigation of the modes of action of the identified active components by various laboratory techniques for the search of new antimicrobials [2,3,4]. Although the exact mechanisms responsible for their antimicrobial activity remain largely unknown, disturbance of bacterial cell membranes by antimicrobial agents has been suggested as one of their mode of actions of plant-derived antimicrobials [5,6,7]. It has also been suggested that the solubility of plant essential oils (EOs) owing to their lipophilic propensity, in the phospholipid of biological membranes plays an important role in their activities by causing membrane damage [8]. Published studies focus on investigating the cellular responses of bacteria that are subjected to non-lethal treatments with potential antimicrobial agents, such as changes in membrane integrity indicated by the leakage of intracellular constituents into the extracellular environment, changes in intracellular pH, and changes in the ability to perform ATP synthesis [2,3,4]. These experiments, however, involve the use of complex living organisms and also because the antimicrobials may induce multiple effects on bacterial cells, the exact molecular mechanisms are therefore not readily defined [9].

On the other hand, studies of thermodynamic properties of bacterial membrane lipids using the Langmuir monolayer system can provide useful information of interactions of antimicrobial compounds with a model membrane at the water–air interface as the lateral packing can be precisely controlled, thus facilitating the measurements of monolayer properties [10,11,12,13,14]. Although the biological membrane is a bilayer, it is believed that many important phenomena which take place in bilayer membrane can be elucidated by experiments on the monolayer at an interface [15]. The Langmuir monolayer technique has been successfully employed to study the characteristics of membrane structure and interaction between lipid and protein molecules, by mimicking both the mammalian and bacterial cell membranes [16]. Monolayers have also been used to evaluate antibacterial peptides, proteins, and more recently chitosan [17,18,19,20], whose antimicrobial properties were extensively reviewed [21]. However, the study of low molecular weight compounds using Langmuir monolayers has not been reported.

In previous studies, we determined the antimicrobial activities (potencies) of a range of natural compounds, including the five being evaluated in the present study, against multiple pathogenic bacteria [2,22,23,24]. In the present study, we evaluated the interaction between the naturally occurring compounds and bacterial membrane lipids on model monolayers composed of bacterial phospholipids, DPPE (zwitterionic lipid), DPPG, and cardiolipin (both anionic lipids), using surface pressure and surface potential isotherms. Gram-negative bacteria are rich in zwitterionic phospholipid in their inner and outer cell membrane, and also contain anionic phospholipid, cardiolipin, while Gram-positive bacteria contain predominantly anionic lipid [25,26]. For instance, the major membrane phospholipids of *E. coli* include 75% PE, 20% PG and 5% cardiolipin [27]. Phospholipids play multiple roles in bacterial cells and are frequently used for mimicking biomembranes. The experimental monolayer technique allowed us to focus on the influence of natural antimicrobials on changes in physical characteristics of the phospholipid monolayers such as surface pressure, surface potential, phase behavior, lipid packing density, and ability of the natural compounds to interact with the phospholipid models.

**Figure 1:**
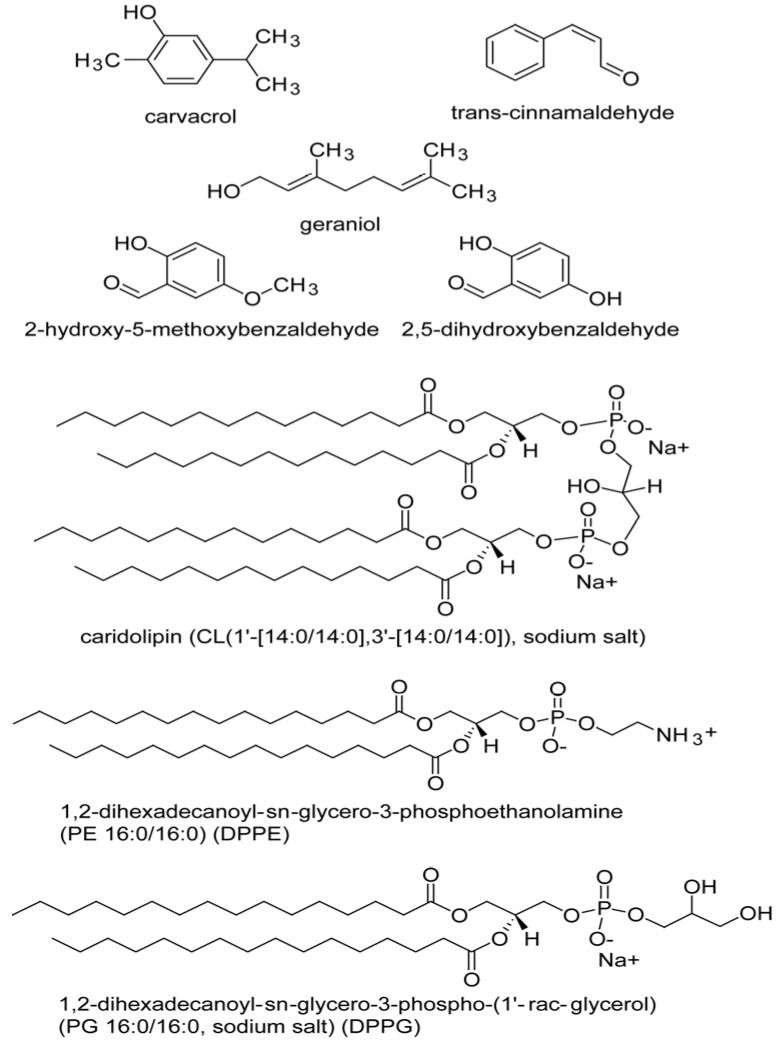
Structures of evaluated five test compounds and three phospholipids.

Studies have been reported on the antimicrobial effect of green tea catechins and black tea theaflavins on virtual cell membranes with the aid of computer modeling [5,28,29]. In the present study, the Langmuir monolayers composed of bacterial phospholipids were used as model membranes to study interactions of the five naturally occurring phenolic compounds (Figure 1) 2,5-dihydroxybenzaldehyde, 2-hydroxy-5-methoxybenzaldehyde, and the plant essential oil compounds carvacrol, cinnamaldehyde, and geraniol, previously found to be active against both Gram-positive and Gram-negative pathogenic microorganisms.

## 2. Results and Discussion

### 2.1. Compression Isotherm and Compressibility Modulus of Monolayer

#### 2.1.1. DPPE Monolayer

**Figure 2:**
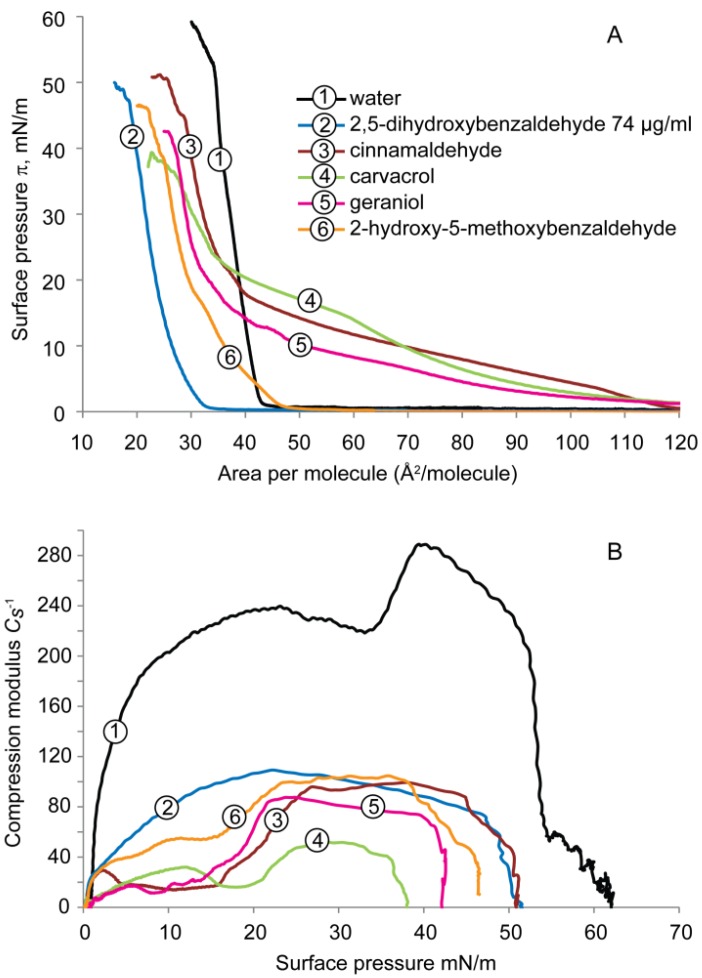
(**A**) Surface pressure-area (π-A) isotherms, and (**B**) compressibility modulus (*C_s_*^−1^) values *versus* surface pressure (π), recorded for the monolayers formed by DPPE on subphases containing pure water with and without antimicrobial compounds.

The surface pressure-molecular area (π-A) isotherms of the DPPE monolayer deposited on subphases containing water with and without test compounds, and recorded during compression, are presented in Figure 2A. 

The values of the *C_s_*^−1^ plotted as a function of the surface pressure are presented in Figure 2B. On pure water subphase, the surface pressure-molecular area isotherm lifts off at ~47 Å^2^/molecule and the surface pressure rises sharply to ~53 mN/m (high slope) and exhibits a typical liquid-condensed (LC) monolayer behavior until it collapses at ~35 Å^2^/molecule (collapse pressure, ~58 mN/m).

Compression isotherms of DPPE recorded on the subphase to which carvacrol, cinnamaldehyde, and geraniol were added showed marked increases in the lift-off values (up to 120 Å^2^/molecule), indicating the expansion of DPPE monolayer with increased molecular area by these compounds. The compression isotherm of DPPE on carvacrol subphase showed a distinct liquid-expanded (LE) to liquid-condensed (LC) transition at surface pressure between 14 and 22 mN/m. The surface pressure of the monolayer rose gradually (shallow slope) until it reached the collapse pressure of ~38 mN/m at ~22 Å^2^/molecule.

With the same lift-off value (~117 Å^2^/molecule), cinnamaldehyde and geraniol displayed a comparable shape of the curves. Both compression isotherms showed extended areas of liquid-expanded phases before turning into the liquid-condensed phase at relatively smaller surface areas (~30–40 Å^2^/molecule), which finally reached the collapse pressure of ~51 and 43 mN/m, respectively. These values are higher than observed with carvacrol but lower than with water. Geraniol did not induce a similar effect as cinnamaldehyde on the DPPE monolayer in the present study.

This data indicates that the addition of these compounds to the subphase expand the isotherm at lower pressures to much greater areas (up to ~120 Å^2^/molecule), suggesting a reduced packing density of DPPE monolayer in the presence of the compounds as compared to water only subphase. Carvacrol had the highest surface pressure (>~20 mN/m) on the onset of condensed phase formation (~23 mN/m) and lower slopes than those of cinnamaldehyde and geraniol. These results indicate the least efficient packing (or most fluidizing) effect of carvacrol on the DPPE monolayer, followed by cinnamaldehyde and geraniol.

For DPPE on 2-hydroxy-5-methoxybenzaldehyde subphase, the lift-off value did not alter compared to water but a decreased collapse pressure of 47 mN/m at a smaller area of 23 Å^2^ per molecule was observed. The surface pressure-area isotherm was characterized by a liquid-condensed monolayer with a diminished formation of LE-LC. When the DPPE monolayer was compressed on a subphase containing 2,5-dihydroxybenzaldehyde at 74 µg/mL, the MIC determined by the previous microbiological study [24], the compression isotherm lifted off at lower molecular area (~33 Å^2^/molecule) compared to water and exhibited a condensed monolayer behavior. The monolayer collapsed at ~15 Å^2^/molecule at a collapse pressure of ~50 mN/m. These results suggest an increased packing density of DPPE molecules in the presence of the antimicrobial.

The compressibility modulus *vs.* surface pressure (*C_s_*^−1^-π), reflecting the interfacial elasticity of the DPPE monolayer is shown in Figure 2B. For pure water, maximal values of *C_s_*^−1^ (240 and 290 mN/m) were observed, which is in agreement with reported findings [18]. The *C_s_*^−1^ values of DPPE monolayers on subphases containing antimicrobials reveal a 2- to 5-fold reduction in the maximum *C_s_*^−1^ values, ranging from 50–108 mN/m. Such a decrease indicates a fluidizing effect of the natural compound or an increased elasticity of the DPPE monolayer because the lower the maximal value for the compressibility modulus, the higher the fluidity/elasticity of the monolayer [30]. Carvacrol (~50 mN/m, 5-fold decrease) exhibited the most significant fluidizing effect, followed by geraniol, cinnamaldehyde, and both 2,5-dihydroxybenzaldehyde and 2-hydroxy-5-methoxybenzaldehyde had the same *C_s_*^−1^ values, at surface pressure of 30 mN/m, the packing pressure found in biological membranes. Overall, the addition of test compounds to the subphase changed the shape of the surface pressure isotherms and decreased the compressibility modulus, reflecting a modification of thermodynamic properties of the DPPE monolayer. Such modifications might be induced by an alteration of the molecular area of the DPPE molecule occupied in the monolayer, a change in the molecular packing effectiveness, and/or a change in the membrane fluidity.

#### 2.1.2. DPPG Monolayer

**Figure 3:**
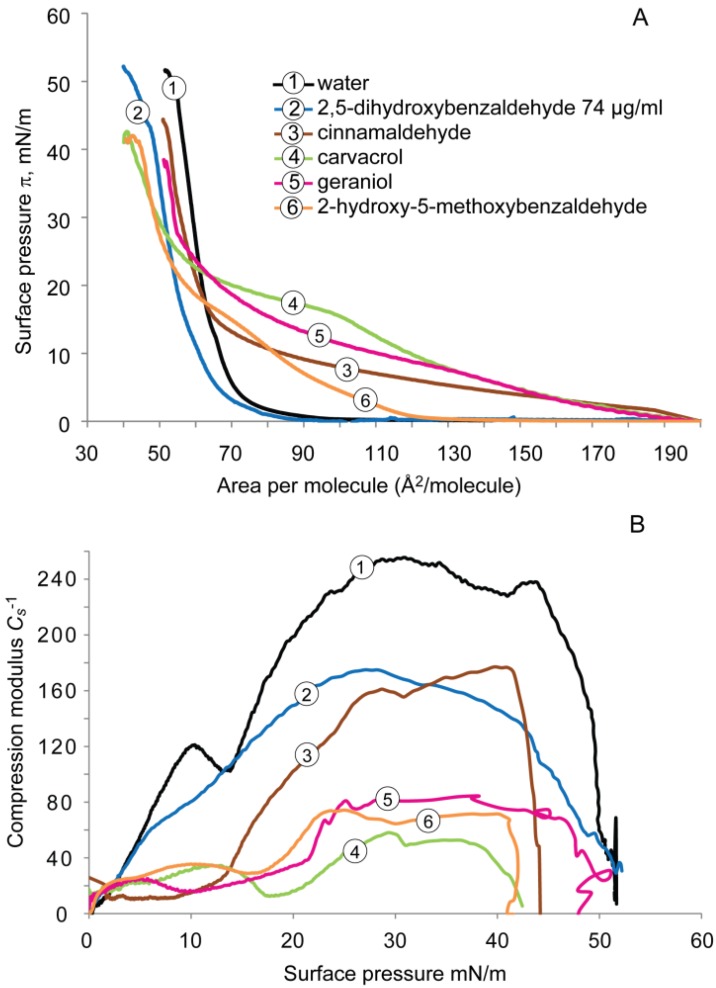
(**A**) Surface pressure-area (π-A) isotherms recorded for the monolayers formed by DPPG on subphases containing water with and without antimicrobials, and (**B**) Compressibility modulus (*C_s_*^−1^) values *versus* surface pressure (π) recorded for the monolayers formed by DPPG on subphases containing water and antimicrobials.

The compression isotherms for the DPPG monolayer deposited on subphases containing water with and without antimicrobials are shown in Figure 3A. For pure water subphase, the lift-off value of DPPG was ~90 Å^2^/molecule and the surface pressure rapidly increased and reached the collapse pressure of ~49 mN/m at 55 Å^2^/molecule. 

Similar to DPPE, surface pressure isotherms of DPPG deposited on the subphase with cinnamaldehyde, carvacrol, or geraniol, caused monolayer expansion, *i.e.*, the lift-off values increased up to 200 Å^2^/molecule and displayed liquid-expanded monolayer behavior with lower slopes than that of water, indicating that the phase transition from liquid-expanded state into the liquid-condensed state was at a lower rate. This observation also suggests a reduced packing and increased fluidity of the DPPG monolayer. The isotherm of DPPG on carvacrol subphase reveals once again a broad two-dimensional phase (LE-LC) transition region between 15 and 22 mN/m with the lowest slope in the liquid-condensed phase. Both compression isotherms of geraniol and cinnamaldehyde presented similar shapes of curves, but the former showed a higher surface pressure (~27 mN/m) at the onset of LE-LC transition than the latter (~14 mN/m), indicating a greater fluidizing effect of geraniol than of cinnamaldehyde. The 2-hydroxy-5-methoxybenzaldehyde subphase increased the lift-off value of DPPG (~125 mN/m) and retained a liquid-expanded phase at large molecular area at 80 Å^2^. Interestingly, 2,5-dihydroxybenzaldehyde displayed a similar surface pressure isotherm to that of water at 74 μL/mL.

The analysis of the elastic area compressibility modulus for DPPG monolayer in the presence of antimicrobials is shown in Figure 3B. On a water subphase, the maximal value of *C_s_*^−1^ was ~255 mN/m at a surface pressure of 30 mN/m. Similar to the DPPE monolayer, all of the compounds caused a significant up to 4-fold reduction in the maximal value of the compressibility modulus ranging from ~60 (carvacrol) to 175 (2,5-dihydroxybenzaldehyde) mN/m compared with water. Carvacrol had the smallest maximal value of *C_s_*^−1^, indicating the greatest fluidizing effect. Similar fluidizing effects on the DPPG monolayer were evident on cinnamaldehyde and 2-hydroxy-5-methoxybenzaldehyde subphases.

### 2.2. Cardiolipin Monolayer

**Figure 4:**
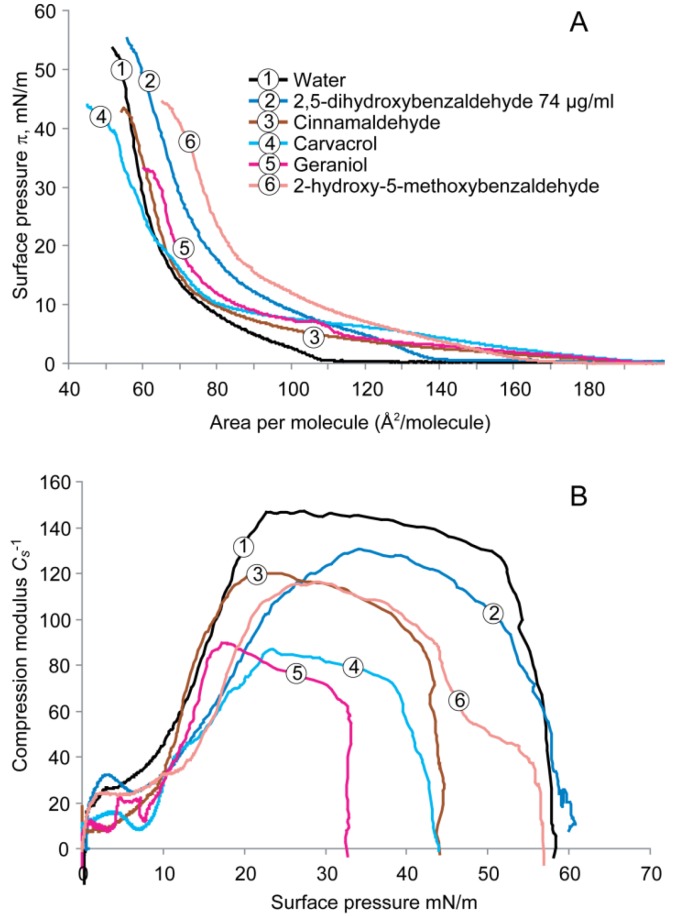
(**A**) Surface pressure-area (π-A) isotherms recorded for the monolayers formed by cardiolipin on subphases containing water with and without antimicrobials, and (**B**) compressibility modulus (*C_s_*^−1^) values *versus* surface pressure (π) recorded for the monolayers formed by cardiolipin on subphases containing water with and without antimicrobials.

The π-A isotherms for the cardiolipin monolayer on subphases containing water with and without antimicrobials are shown in Figure 4A. On water subphase, the isotherm lifted off at ~110 Å^2^/molecule and the surface pressure rose gradually into the LC phase up to ~53 Å^2^/molecule. The monolayer then collapsed at a pressure of ~52 mN/m.

The antimicrobials increased the lift-off value to 140–180 Å^2^/molecule and stabilized the liquid expanded phase over large areas at low pressures. These compounds reduced the lipid packing and expanded the cardiolipin monolayer. Carvacrol, geraniol, and cinnamaldehyde showed higher lift-off values than did 2,5-dihydroxybenzaldehyde or 2-hydroxy-5-methoxybenzaldehyde.

Figure 4B shows the interfacial moduli of the area compressibility (*C_s_*^−1^) of the cardiolipin monolayer on subphase of antimicrobials. On the water subphase, the maximal value of *C_s_*^−1^ was ~150 mN/m at ~20 mN/m. The *C_s_*^−1^ value dropped slightly to ~145 mN/m at 30 mN/m. All compounds caused a decrease in the maximal value of compressibility modulus from ~85 to 130 mN/m compared with that of water. Carvacrol showed the strongest fluidizing effect, followed by geraniol as the second most effective compound.

### 2.3. Comparison of Compression Isotherms of the Three Phospholipid Monolayers

Lipid monolayers were formed at the air–liquid interface using DPPE, DPPG, and cardiolipin to mimic the surface of the bacterial membranes. A typical isotherm of lipid monolayer on water subphase consists of three regions [31]. Initially, the monolayer behaves as a two-dimensional gas, the so-called liquid-expanded (or gaseous stage), where the lipid molecules are far apart and do not to interact with each other. When the area of the monolayer is reduced by film compression, the lipid molecules become closer and start to interact with each other at the liquid state. With further compression, the lipid film molecules become tightly packed between lipid heads at the liquid-condensed or solid state and then become vertically oriented with lipid tails towards the air, with the hydrophilic heads oriented to the water subphase. On further compression, collapse of monolayer occurs. Interaction between antimicrobials and monolayer films influences the arrangement of lipid molecules and the surface pressure-area (π-A) isotherm of the monolayers.

Considering the π-A isotherms compared to water, four out of the five antimicrobials caused significant modifications in the isotherms of the three model monolayers in comparison to the phospholipids deposited on pure water. The increase in the lift-off value indicates that the monolayers occupied a larger area at low surface pressure on natural compound subphases, indicating a decrease in lipid molecular packing and thus more fluidity of the monolayers. This means that the antimicrobials might have been incorporated into the liquid phase (LE and/or LC) of the lipid monolayer, thus modifying the structure of the lipid matrix.

This opposite effect of 2,5-dihydroxybenzaldehyde on the zwitterionic DPPE monolayer may be explained by the interactions between the two phenolate anions (*i.e.*, the dissociated and negatively charged phenolic hydroxyl groups) of the compound and the positively charged amino group of the lipid through electrostatic force which might draw the lipid molecules more closer (*i.e.*, one molecule linking two lipid molecules and pulling them toward the liquid subphase), thus increasing the lipid packing. It slightly condensed the anionic DPPG monolayer but increased the elasticity of the lipid. The former may due to a reduced hydrocarbon chain mobility, suggesting the penetration of the compound into the hydrophobic region of the lipid due to the lipophilic attraction, while the latter may be explained by the repulsion between the compound and the negatively charged lipid headgroup, as well as between the adjacent lipid molecules. It behaved different on cardiolipin monolayer which is also an anionic lipid. It expanded the monolayer and decreased lipid packing, which may be due to a stronger electrostatic repulsion between the polar lipid heads (with two negatively charged phosphate groups and one hydroxyl group) and hydroxyl groups of the compound.

The factor that determines compressibility of the isotherm is its slope. The changes in the compressibility modulus reflect the physical state of the lipid monolayer compressed on different subphases. The higher the value of compressibility modulus, the higher the rigidity of the model membrane. A low value indicates a high fluidity of the model membrane [26]. In the present study, all test compounds lowered the compressibility modulus thus increasing the elasticity of the monolayers, with the largest effect found on the DPPE monolayer.

The antimicrobials might have modified the lipid monolayer structure by incorporating into the lipid monolayer, generating an aggregation of antimicrobials and lipids, thus increasing the membrane fluidity. A decreased compressibility modulus and a lift-off at larger molecular area reflect a greater intermolecular interaction between lipid and water through bridging of the natural compound at the air-water interface. Other investigators also found that some antimicrobial molecules seem to have a greater impact on the lipid molecules at the gas or liquid phases of the monolayer than at the solid phase [27].

The decrease in the lift-off value at low surface pressure reflects a better packing effectiveness of lipid monolayer by the natural compounds. Considering the change in the lift-off value, addition of 2,5-dihydroxybenzaldehyde to the subphase at low concentration (74 μg/mL) caused a decrease (DPPE), no change (DPPG), and an increase (cardiolipin), whereas 2-hydroxy-5-methoxybenzaldehyde did not alter DPPE but did induce an increase on both DPPG and cardiolipin monolayers.

These results suggest that the two structurally similar compounds may undergo different interactions with the lipid molecules, although both compounds inactivated multiple pathogens [2]. In fact, 2,5-dihydroxybenzaldehyde has one more OH group on its benzene ring and has a distribution coefficient (Log D) of 1.10 at neutral pH (7.4), whereas one phenolic group is replaced by a methoxyl group (CH_3_) in 2-hydroxy-5-methoxybenzaldehyde, which increases its Log D (pH 7.4) to 1.50, rendering it more lipophilic. It seems that the hydrophilic and ionic properties associated with the two phenolic OH groups and the lipophilic properties associated with the benzene moiety facilitate interactions with the monolayer.

### 2.4. Surface Potential of the Monolayer

#### 2.4.1. DPPE Monolayer

Molecules near the liquid surface have a specific orientation. Spreading a monolayer on a clean water surface will produce a change in the orientation at the interface, so called the surface potential, which arises from the dipole moments of the film-forming materials, the change in orientation of head or tail group in the lipid monolayer and the water molecules in the subphase. Surface potential measurements can therefore provide information regarding the orientation of the film constituents [10,31]. The value of surface potential rises from the dipole moment of both the lipid molecules and water and other molecules in the subphase during compression.

**Figure 5:**
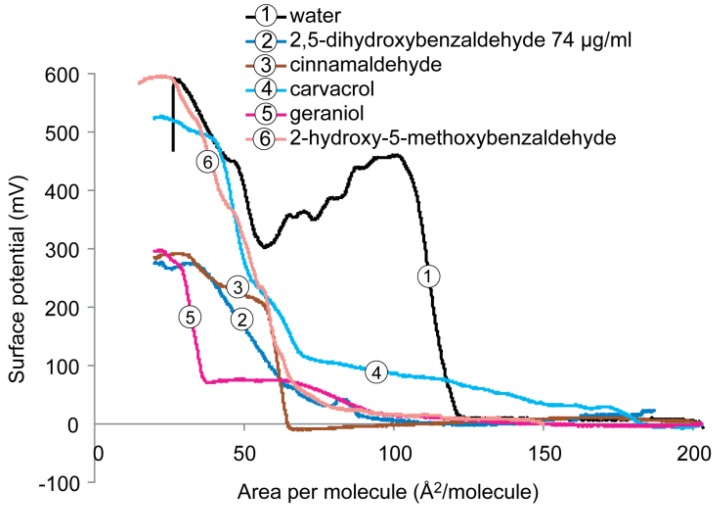
Surface potential *versus* molecular area recorded for the monolayers formed by DPPE on subphases containing water with and without antimicrobials.

Surface potential isotherms of DPPE monolayer on a subphase containing pure water with and without antimicrobials are shown in Figure 5. Considering the monolayer of DPPE at the condensed state (pressure ~30 mN/m and area per molecule ~30–40 Å^2^/molecule), it was found that the addition of 2-hydroxy-5-methoxybenzaldehyde did not change the maximum surface potential as it was ~600 mV, the same value as the DPPE on pure water subphase. Addition of carvacrol to water subphase slightly lowered the surface potential to ~520 mV. This may be explained by the interactions between carvacrol and the zwitterionic lipid molecules, *i.e.*, between the phenolic hydroxyl group of the compound and the protonated amine (NH_3_^+^) of the lipid headgroup via the formation of a hydrogen bond, as well as the hydrocarbon ring of the compound with the hydrocarbon chain of the lipid tail through hydrophobic attraction.

A significant reduction in the maximum surface potential value (~300 mV, 50% decrease) of DPPE was found in the presence of 2,5-dihydroxybenzaldehyde, cinnamaldehyde or geraniol. The decrease of surface potential may be due to the induced dipolar interactions upon compression of the monolayer between the antimicrobial and lipid molecules and water molecules, resulting in the formation of antimicrobial-lipid complexes (aggregates), and a decrease in dipole density of the DPPE monolayer.

#### 2.4.2. DPPG Monolayer

**Figure 6:**
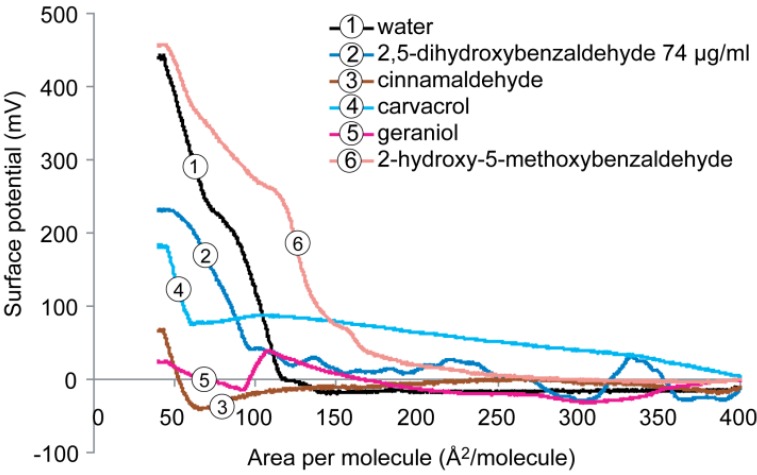
Surface potential *versus* molecular area isotherms recorded for the monolayers formed by DPPG on subphases containing water with and without antimicrobials.

Figure 6 shows the behavior of the surface potential-area isotherm of the DPPG monolayer on subphase containing water with and without antimicrobials. When surface potentials are compared at comparable surface pressure and area (pressure = ~30 mN/m and area = ~40–80 Å^2^/molecule), similar to DPPE, the addition of 2-hydroxy-5-methoxybenzaldehyde to the water subphase caused a slightly increased surface potential compared DPPG on pure water subphase (~450 mV). In contrast, the other four compounds caused a significant decrease in the maximum surface potential. In the presence of 2,5-dihydroxybenzaldehyde and carvacrol, the maximum surface potential dropped from 450 mV to ~230 and ~180 mV, respectively. Cinnamaldehyde and geraniol induced a significant reduction up to 95% to ~75 and ~25 mV, respectively, suggesting the formation of domains in the anionic phospholipid DPPG monolayer. The marked reduction of surface potentials was a result of the combination of repulsion between phospholipid molecules (negatively charged lipid headgroup) and interaction between the more hydrophobic compounds (*i.e.*, cinnamaldehyde and geraniol) and the hydrocarbon chains of the lipid. In the latter case, the hydrophobic attraction may pull the lipid tails toward the liquid surface thus reducing the thickness of the monolayer due to the change in the orientation of the lipid tail chain which is usually perpendicular upon compression, as well as the dipole-dipole attraction between the lipid molecules and water molecules.

### 2.5. Cardiolipin Monolayer

**Figure 7:**
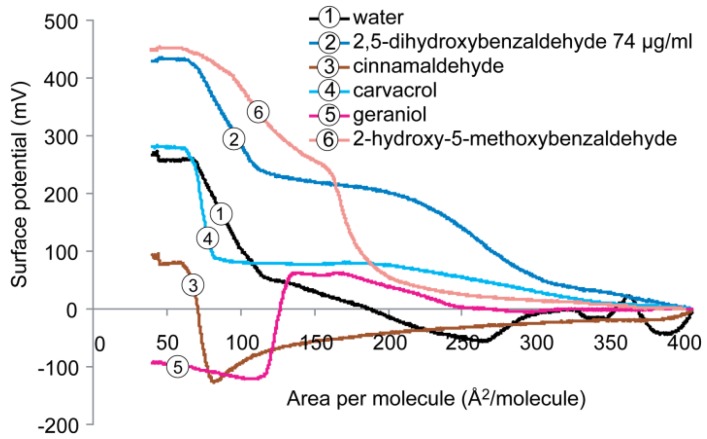
Surface potential *versus* molecular area isotherms recorded for the monolayers formed by cardiolipin on subphases containing water with and without antimicrobials.

The surface potential isotherms of the cardiolipin monolayer on subphase containing water with and without antimicrobials are presented in Figure 7. On pure water subphase, the maximum surface potential of cardiolipin monolayer was ~260 mV, being the lowest in the three monolayers tested. At comparable conditions of surface pressure and molecular area (pressure ~30 mN/m and area per molecule ~50–100 Å^2^/molecule), additions of 2,5-dihydroxybenzaldehyde and 2-hydroxy-5-methoxybenzaldehyde greatly increased the surface potential to ~430 and 450 mV, respectively. Addition of carvacrol did not change the maximum surface potential of the cardiolipin monolayer. Similar to DPPG monolayer, both cinnamaldehyde and geraniol significantly lowered the surface potential of cardiolipin monolayer to ~90 mV (70% reduction). The negative value observed for geraniol suggests that changes occurred in the orientation of the lipid molecules adjacent to the water subphase which had rotated by 180° as well changes in the charge distribution of the monolayers.

### 2.6. Mechanistic Aspects

Surface potential arises from the total dipole moment of a monolayer system from three components; the dipole moment at the hydrophobic region (lipid tail), the polar region of the lipid molecules (head), and the aqueous subphase adjacent to the lipid molecules [31]. It is not possible to measure any of the above parameters individually but only the total surface potential of the monolayer. Moreover, the dipole moment within the antimicrobials, the monolayer, and the hydrophobic thickness can also influence the total dipole moment. Modification of membrane fluidity could be a consequence of these individual or combined factors. The higher the fluidity of the monolayer, the lower the dipole density. Lower surface potentials also parallel to a lower total dipole moment.

The ability of natural antimicrobials to modify the surface potential of monolayer of DPPE, DPPG, and cardiolipin observed in the present study may due to two possible mechanisms: (a) the modification of lipid hydration of the monolayer through the reorientation of water molecules just below the polar heads by addition of the natural compounds; and/or (b) the modification of the monolayer thickness by hydrophobic attraction at the lipid tail region between the hydrocarbon chains, thus reducing the packing effectiveness of the lipid molecules in the monolayer. Considering the π-A isotherms obtained from the three lipid monolayers compressed on subphase containing water only, DPPE displayed the highest molecular packing efficiency, followed by DPPG then cardiolipin. This may due to the stronger interactions between the zwitterionic polar headgroups of the adjacent lipid molecules, *i.e.*, the phosphate group and the protonated amine group, than the van der Waals forces between the charged lipid head groups and the water molecules. In contrast, both anionic phospholipid DPPG and cardiolipin displayed lower packing density than that of DPPE, which may due to the electrostatic repulsion between their negatively charged polar heads which will not allow the lipid molecules to pack as tightly as DPPE. Since the area per molecule determines the dipole density of the monolayer, these observations explain why DPPE has both the highest packing density and surface potential.

Addition of the natural compounds to the subphase either decreased or increased the surface potential of the monolayers of DPPE, DPPG, and cardiolipin. Cinnamaldehyde and geraniol induced the biggest reduction of surface potential in all three monolayers which could be ascribed by the strong hydrophobic attraction between the aromatic part or hydrocarbon chain of the compounds and the phospholipid acyl chains [32]. Among the five tested compounds except carvacrol, these are the two most hydrophobic compounds with only one functional group (*i.e.*, hydroxyl in geraniol and carbonyl in cinnamaldehyde). The LogP values are 2.12 and 3.29 for cinnamaldehyde and geraniol, respectively. Cinnamaldehyde has been reported to inhibit growth of both Gram-positive and Gram-negative organisms and geraniol was found to inhibit *E.* *coli* only at relatively high concentration [6,22]. The present study showed that geraniol could reduce the surface potential of the DPPE monolayer to a similar extent as cinnamaldehyde. Elsewhere, it was reported that cinnamaldehyde inhibits pathogens by a different mechanism than the other antimicrobials [6,33].

Carvacrol caused a significant reduction of surface potential in DPPG, a slight reduction in DPPE but did not induce any change in cardiolipin monolayer. It seemed to be the most effective compound which caused the largest expansion (Figure 2A and Figure 3A) and highest elasticity (Figure 2B and Figure 3B) of both DPPE and DPPG monolayers. The effect of carvacrol on DPPE monolayer may be ascribed by the penetration of the compound (LogP = 3.28) into the lipid molecules through hydrocarbon attraction at the lipid tail region and the hydrogen bonds between the phenolic hydroxyl group and the lipid headgroup.

The phenolic OH group of carvacrol has features a pKa value of ~10.9 and only some 0.1% of molecules are dissociated at neutral pH and can form mostly hydrogen bonds with lipid headgroups and water molecules. In the case of DPPG, the reduction in surface potential by carvacrol suggested a change in orientation of head and/or tail group of the lipid molecules which may due to the repulsion between the negatively charged phosphate groups as well as the hydrocarbon attraction between the aromatic hydrocarbon of the carvacrol and phospholipid acyl chains, thus reducing the thickness of the lipid monolayer. Moreover, the addition of carvacrol may rearrange orientation of the water molecules in the subphase due to the hydrogen bonding between the phenolic hydroxyl group and water molecules, thus lowering the total dipole moments. This may also result in the hydration of the lipid monolayer when the water molecules are attracted to the lipid monolayer. Ultee *et al.* found that the phenolic OH group of carvacrol was essential for action against the pathogen *Bacillus cereus* [34]. Similar observations were reported by Friedman based on studies of the effect of sublethal concentrations of carvacrol on the autofluorescence of *E.* *coli* bacteria [35]. Other investigators concluded that the OH group of carvacrol is not essential but does have special features added to the antimicrobial mode of action of carvacrol [36]. These observations and the results of the present study indicate that the phenolic OH group of carvacrol is a key structural feature of the molecule that is involved in both antimicrobial activity against foodborne pathogens via disruption of cell membranes and afinity to the monolayer model membrane.

Addition of 2,5-dihydroxybenzaldehyde reduced the surface potential of both DPPE and DPPG but increased the surface potential of cardiolipin monolayer, whereas the presence of 2-hydroxy-5-methoxybenzaldehyde did not change the surface potential of both DPPE and DPPG but increased its value on cardiolipin monolayer. The OH groups of 2,5-dihydroxybenzaldehyde and 2-hydroxy-5-methoxybenzaldehyde are more acidic with pKa values around 8.5. Thus, a significant fraction of 2,5-dihydroxybenzaldehyde and 2-hydroxy-5-methoxybenzaldehyde are dissociated at neutral pH and can also produce electrostatic interaction with the lipid headgroups of the model membranes.

The results of the present study confirmed the importance of the number as well as position of the phenolic hydroxyl group for their effects on cell membranes as they are the only structural difference between these two compounds. The two phenolic hydroxyl groups render the compound more effective towards both the lipid and water molecules in DPPE, reflecting by the higher packing density (Figure 2A), higher elasticity (Figure 2B) and higher fluidity (Figure 5). When the 5-OH is replaced by a methoxyl group (OCH_3_) as in the case of 2-hydroxy-5-methoxybenzaldehyde, it caused no change in the parking density and insignificant change in the surface potential.

The effects of phenolic OH groups on disruption of cellular membranes have also been demonstrated for pathogenic bacteria [6]. For example, Sirk *et al.* concluded on the basis of computer simulations using a model cell membrane (1-palmitoyl-2-oleoylphosphatidylcholine, POPC) that green tea catechins and black tea theaflavins could penetrate the virtual bilayer membrane by forming strong hydrogen bonds between OH groups of the tea compounds and oxygen atoms of the lipid head-groups, thus initiating the disruption of cell membranes in living cells [5,28,29]. Table 1 summarizes the quantitative experimental values for the five test compounds. 

**Table 1:** Summary of experimental thermodynamic parameters for the interactions of five test compounds with three different phospholipid monolayers.

Monolayer Parameter	Test Substance					
	Water	Carvacrol	Cinnamal-dehyde	Geraniol	2,5-Dihydroxy-benzaldehyde	2-Hydroxy-5-methoxy-benzaldehyde
DPPE						
lift-off value (Å^2^/molecule)	43	120	117	120	33	43
collapse (mN/m)pressure	58	38	51	43	50	47
compressibility-surface pressure (*C_s_*^−1^)	240	50	95	88	108	100
surface potential (mV)	600	520	300	300	300	600
surface dipole moment area (Å^2^/molecule)		185	75	100	90	75
DPPG						
lift-off value (Å^2^/molecule)	90	200	200	200	90	125
collapse (mN/m)pressure	49	42	44	38	52	42
compressibility-surface pressure (*C_s_*^−1^)	255	60	175	80	170	72
surface potential (mV)	450	180	75	25	230	460
surface dipole moment area (Å^2^/molecule)	125	400	250	180	270	250
Cardiolipin						
lift-off value (Å^2^/molecule)	110	180	180	180	140	180
collapse (mN/m)pressure	52	39	43	32	52	44
compressibility-surface pressure (*C_s_*^−1^)	145	85	120	90	130	115
surface potential (mV)	270	280	80	−100	430	450
surface dipole moment area (Å^2^/molecule)	260	400	400	250	320	210

It is also relevant to note that the monolayer technique has been used in recent research [7,37] to study the mode of action of antimicrobial cationic peptides. The peptides are attracted to anionic lipids and can attach to their phospholipid head group, causing reorganization of membrane lipid molecules and membrane disruption, resulting in increased membrane permeability [26]. By comparison, due to a high degree of hydrophobicity of the aromatic hydrocarbon and hydrogen bonding of the phenolic hydroxyl groups, as well as the hydrophilicity of the dissociated phenolate anion towards the lipid headgroups of the membrane, essential oil compounds can partition into the hydrophobic region of fatty acyl chains of the membrane, thus changing the packing, fluidity the thickness of the lipid layer. This event disrupts the cell membrane and leads to the death of the bacteria.

## 3. Experimental

### 3.1. Materials

1,1',2,2'-Tetratetradecanoyl cardiolipin sodium salt (cardiolipin), 1,2-dihexadecanoyl-*sn*-glycero-3-phosphoethanolamine (DPPE), and 1,2-dihexadecanoyl-*sn*-glycero-3-phospho-(1'-*rac*-glycerol) (DPPG) were purchased from Avanti Polar Lipids (Alabaster, AL, USA). Stock solutions of the lipids (5 mg/mL) were prepared in chloroform (Sigma, Dorset, UK) and stored at −20 °C. Reverse osmosis water (Milli Q water system; Millipore, Molsheim, France) was used in all experiments. The following test compounds were obtained from Sigma (St. Louis, MO, USA): 2,5-dihydroxybenzaldehyde, 2-hydroxy-5-methoxy-benzaldehyde, carvacrol, cinnamaldehyde, and geraniol. The purity of these compounds ranged from 98% to 99.9%. The test compounds were prepared as stock solutions in reverse osmosis water for 2,5-dihydroxybenzaldehyde (100 mg/mL) and in chloroform for the other four compounds (500 mg/mL). The stock of the powder compound was freshly prepared before each experiment and used on the same day. The stocks of other compounds were stored at −20 °C.

Subphases were prepared as follows: stock solution of 2,5-dihydroxybenzaldehye was injected into the water subphase to give a final concentration of 74 µg/mL, which was the minimum inhibitory concentration (MIC) determined in a previous antibacterial study [24]. For the other compounds, 10 µL of the stock solution (500 mg/mL) was injected into the water subphase to make the final concentration of 0.5 mg/mL.

### 3.2. Monolayer Measurements

A Langmuir trough (µTrough XL with Precision Plus Trough, Kibron, Helsinki, Finland) equipped with a computer–controlled microbalance (Kibron) and MicroSpot (Kibron) was used to measure surface pressure–area (π-A) and surface potential–area (Δψ-A) isotherms, using the embedded features of the control software (FilmWare 3.61; Kibron). The surface area of the trough was 227.15 cm^2^ and the volume of the subphase was 100 mL. Experiment was repeated twice.

The lipids prepared in chloroform were deposited on the surface of water subphase using a 5-µL Hamilton micro-syringe (Supelco, Bellefonte, PA, USA). Water was used as the negative control. The selection of the five natural compounds for evaluation was based on our previously reported antimicrobial assays with these same compounds and geraniol was served as a control compound in this study as it was not active in the previous study [24]. After a 10 min equilibrium to ensure evaporation of solvent, film compression was started by moving the two barriers symmetrically. The compression rate of 21.157 Å^2^/chain/min allowed reorientation and relaxation of the lipids. Surface pressure (π), measured (±0.1 mN/m) using a metal wire probe (Kibron) linked with a high precision microbalance connected to a computer, is defined as π = γ_0_ − γ, where γ_0_ is the surface tension of the water and γ is the surface tension in the presence of a lipid monolayer.

Surface dipole potential (ψ) was measured (±0.1 mV) using the vibrating plate method (MicroSpot; Kibron). All isotherms were recorded at 23 °C. The subphase temperature was controlled thermostatically to within 0.1 °C by a circulating water system (Grant Instruments Ltd., Cambridgeshire, UK).

### 3.3. Analysis of Isotherms

The value for monolayer isothermal compressibility (*C_s_*^−1^) for the indicated film compositions at the given surface pressure (π) was obtained from π-A data as follows:

*C_S_*^−1^ = −A (∂π/∂A), where A is the area per molecule at the indicated surface pressure π. The analysis of *C_S_*^−1^ can be used to identify the phase transition points and to facilitate comparisons of compression modulus values at surface pressures around 30–35 mN/m; the pressures found in biological membranes. The higher the value of *C_s_*^−1^, the lower the interfacial elasticity [17]. To remove background noise, the adjacent averaging smoothing method with 50 points of window was performed using Origin 8 Data Analysis and Graphing software (OriginLab^®^ Corporation, Northampton, MA, USA).

## 4. Conclusions

The present study complements the above-mentioned related molecular dynamics studies on the interactions and binding of antimicrobial green tea catechins and black tea theaflavins to biological membranes [5,28,29]. Our results suggest that natural antimicrobial compounds modify the bacterial cell membrane structure by incorporating into the lipid monolayer, forming aggregates of antimicrobials and lipids, reducing the packing effectiveness of the lipid molecules, increasing the fluidity of the membrane, and altering the dipole moment of the monolayer. These events are strongly influenced by the structures of the antimicrobials and the nature of the monolayer. The detailed experimental data obtained indicate that the evaluated antimicrobials directly target and disturb the structures of “common” phospholipids of bacterial cell membranes. Unlike simulation studies, our conclusions are based on experimental data. The present and previous cited studies offer insights into the interaction between low molecular weight antimicrobials compounds and surfaces of membranes and can help identify bioactive health-promoting compounds for use in healthcare, human foods and animal feeds. The present study complements and extends numerous related studies on the interaction of bioactive compounds with model membranes evaluated in the present study, including cardiolipin-containing membranes [38,39] and DPPE- and DMPG-based membranes [40,41,42,43].

## Abbreviations

*C_s_*^−1^: isothermal compressibility of monolayer
*C_s_*^−1^-π: compressibility-surface pressure of monolayer
DPPE: 1,2-dihexadecanoyl-*sn*-glycero-3-phosphoethylamine
DPPG: 1,2-dihexadecanoyl-*sn*-glycero-3-phospho-(1'-*rac*-glycerol)
LC: liquid-condensed
LE: liquid-expanded
LE-LC: two-dimensional phase transition
LPS: lipopolysaccharide
LTA: lipoteichoic acid
MIC: minimum inhibitory concentration
POPC: 1-palmitoyl-2-oleoylphosphatidylcholine
π: surface pressure of monolayer
πA: surface pressure molecular area of monolayer
